# Adaptation of the parasitic plant lifecycle: germination is controlled by essential host signaling molecules

**DOI:** 10.1093/plphys/kiaa066

**Published:** 2020-12-18

**Authors:** Harro Bouwmeester, Changsheng Li, Benjamin Thiombiano, Mehran Rahimi, Lemeng Dong

**Affiliations:** Plant Hormone Biology group, Green Life Sciences cluster, Swammerdam Institute for Life Science, University of Amsterdam, 1098 XH Amsterdam, The Netherlands

## Abstract

Parasitic plants are plants that connect with a haustorium to the vasculature of another, host, plant from which they absorb water, assimilates, and nutrients. Because of this parasitic lifestyle, parasitic plants need to coordinate their lifecycle with that of their host. Parasitic plants have evolved a number of host detection/host response mechanisms of which the germination in response to chemical host signals in one of the major families of parasitic plants, the Orobanchaceae, is a striking example. In this update review, we discuss these germination stimulants. We review the different compound classes that function as germination stimulants, how they are produced, and in which host plants. We discuss why they are reliable signals, how parasitic plants have evolved mechanisms that detect and respond to them, and whether they play a role in host specificity. The advances in the knowledge underlying this signaling relationship between host and parasitic plant have greatly improved our understanding of the evolution of plant parasitism and are facilitating the development of more effective control measures in cases where these parasitic plants have developed into weeds.

## Teaser

Root parasitic plants grow on the roots of other plants and germinate only in the presence of that host, on which they completely depend, through the perception of host presence signaling molecules called germination stimulants.

**Outstanding questions**

- Have we overlooked the role of germination stimulants in facultative parasites?
- What is the biological relevance of the observation that many plant species produce and secrete a range of different strigolactones?
- Have parasitic plants evolved mechanisms to compensate for low phosphorus availability, a condition that stimulates their germination?
- What is the contribution of the HTL strigolactone receptors to host specificity in parasitic plants or does downstream signaling play a role?
- What other, nonstrigolactone, germination stimulants can parasitic plants respond to and does this require adaptation in the HTL receptors?
- What is the role of germination and underlying mechanism in the rapid adaptation of (orobanchaceous) parasitic plants to a new host?

## Introduction

Parasitic plants rob all or a large part of the water, assimilates, and nutrients that they need for growth and development from the host on which they grow, making many of them important agricultural weeds (Hearne, 2009; Parker, 2012; Rodenburg et al., 2016). This parasitic lifestyle requires a close coordination with the lifecycle of the host. This holds especially true for the parasitic plants of the Orobanchaceae, such as *Alectra* and *Striga* spp. (witchweeds) and *Orobanche*, and *Phelipanche* spp. (broomrapes) that are completely dependent on a host for survival. Hereto, they have evolved a number of host detection/host response mechanisms of which the germination in response to chemical host signals is critically important. This phenomenon was first discovered in the mid-1900s (Brown et al., 1949). In this update review, these germination stimulants are discussed, including how ubiquitous they are and to which chemical classes they belong. An intriguing question is whether these signals convey specificity to the host parasite relationship and—with an emphasis on the most important class of germination stimulants, the strigolactones—what determines their reliability as host presence signals. Related to this is the question of why hosts produce germination stimulants. This is discussed in relation to the fact that the germination stimulants have other, beneficial, roles for the host producing them. One of the most astounding adaptations that parasitic plants evolved is the capacity to repurpose the latter signals as germination stimulants. This is reviewed elsewhere in this issue (see Nelson et al., this issue). Here, we focus on the role these receptors may play in host specificity of parasitic plants.

## The lifecycle of parasitic plants

**Figure 1:**
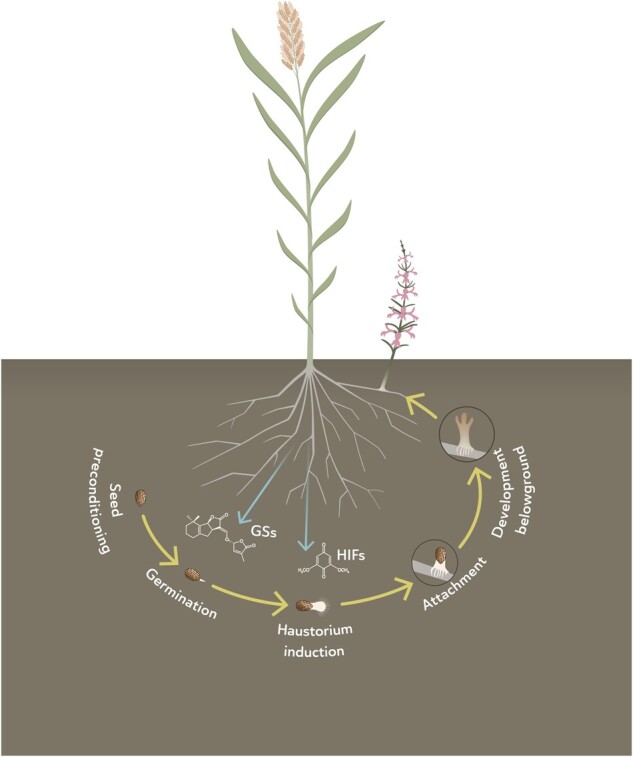
Lifecycle of root parasitic plants. Seed dormancy release (usually called preconditioning) occurs when exposed to the proper environmental conditions (warm temperature and high moisture). Seed germination occurs upon detection of host germination stimulants by nondormant seeds. Seedlings develop haustoria when exposed to haustorium-inducing factors. The haustorium establishes a connection with the host vasculature, after which a seedling develops that grow belowground for several weeks until emergence. The emerged parasite develops aboveground, flowers, and produces seeds that contribute to the seed bank.

Parasitic plants produce large numbers of seeds that—similar to nonparasitic, wild, angiosperms—are dormant when shed from the mother plant. Under favorable temperature and moisture conditions, dormancy is released, which sensitizes the seeds towards their germination stimulant (Matusova et al., 2004; Figure 1). Although the mechanism underlying these changes in dormancy is still not completely understood, gibberellic acid and abscisic acid (ABA) seem to play important roles (Yao et al., 2016b; Bao et al., 2017). While nonparasitic angiosperms require factors such as light and an optimal temperature for induction of germination after dormancy release, parasitic plant seed germination requires the presence of a chemical signal indicative of the vicinity of a host, called a germination stimulant (Matusova et al., 2004; Figure 1). In the absence of a germination stimulant, the seeds will gradually resume their dormant state (Matusova et al., 2004; Song et al., 2005). One to 2 d after perception of the germination stimulant, the radicle emerges from the seed, reaching a length of a few millimeters up to 1 cm. Perception by the parasite of host-derived compounds, called haustorium-inducing factors, subsequently results in the formation of a special invasive organ, the pre-haustorium, characterized by the swelling of the radicle tip and proliferation of haustorial hairs on the surface (Cui et al., 2018; Goyet et al., 2019; Wada et al., 2019; Figure 1). Upon contact with the host root, the prehaustorium develops intrusive cells that penetrate the root forming the haustorium, a physiological bridge between the vascular system of the parasite and that of the host through which the parasite withdraws water and nutrients (Losner-Goshen, 1998; also see Yoshida et al. in this issue). In witchweeds, this connection consists of a xylem–xylem connection while broomrapes establish connections with both the phloem and the xylem (Westwood, 2013). The connection may fail, which is called post-attachment resistance or incompatible interaction, and may be due to a hypersensitive reaction, cell death, and the accumulation of phenolic compounds, among others (Cissoko et al., 2011; Huang et al., 2012; Mutuku et al., 2019). In the case of a compatible attachment, the parasite further develops belowground for a few weeks before emerging from the soil. The lifecycle is then completed with a vegetative phase, flowering, and seed production (Figure 1).

## What are germination stimulants?

Germination stimulants trigger the germination of obligate root parasitic plants of the *Alectra*, *Striga*, *Orobanche*, and *Phelipanche* genera. Facultative root parasites of the Orobanchaceae such as *Rhinantus* and *Triphysaria* spp. germinate in water and do not seem to require a germination stimulant. The latter also holds for seeds of parasitic plants from other families such as mistletoes and *Cuscuta* spp. Germination stimulants are identified using a bioassay: after a dormancy releasing treatment, parasite seeds are incubated with a root exudate or pure compounds and germination is evaluated (Box 1). Bioassay-guided fractionation can be used to elucidate the chemical nature of the germination stimulant after which analytical methods can be used for germination stimulant quantification (Box 1; Cook et al., 1972; Sato et al., 2003, 2005; Flokova et al., 2020). The first class of germination stimulants to be discovered was the strigolactones (Box 2; Table 1; Cook et al., 1972; Butler, 1995). Since their discovery, compounds from many other chemical classes have been isolated from root exudates of parasitic plant hosts or microorganisms and shown to display a certain level of witchweed (*Striga* spp.) and/or broomrape (*Phelipanche* and *Orobanche* spp.) seed germination inducing activity in vitro. However, for many such compounds there are no indications that they play a role in vivo.

**Figure 2:**
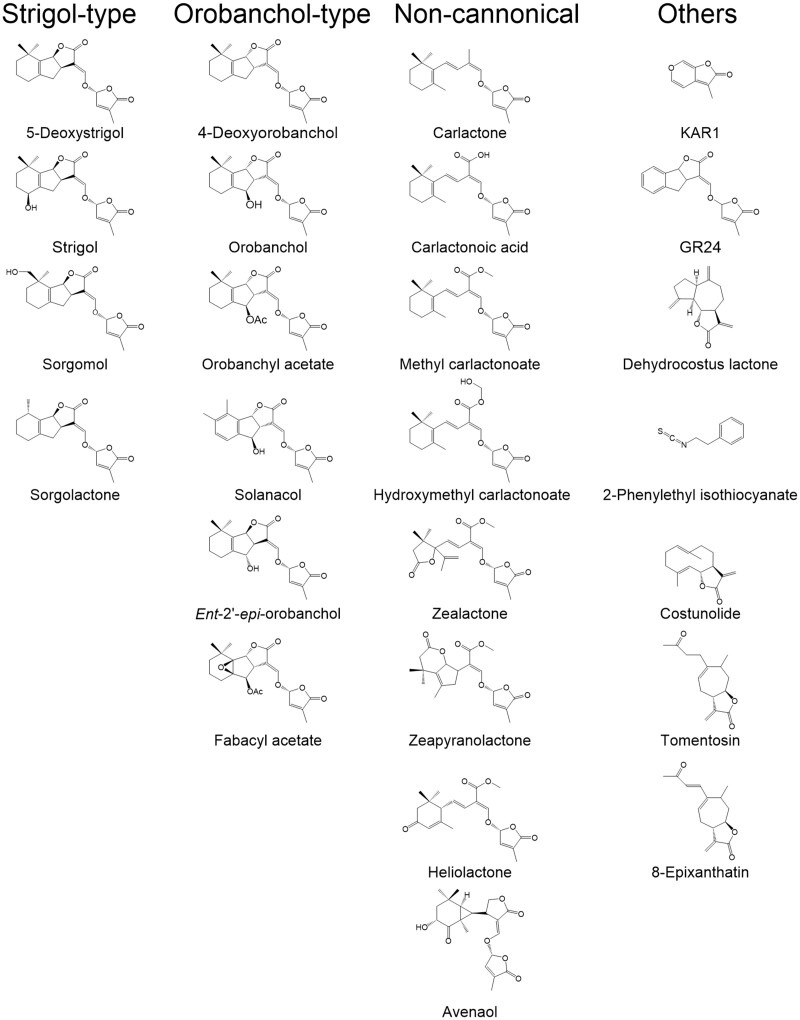
Structures of root parasitic plant germination stimulants.

The strong stimulation of strigolactone exudation by low phosphorus availability suggests that parasitic plant infection is higher under conditions of low phosphorus availability (Yoneyama et al., 2007a; Jamil et al., 2011, 2012b, 2013). This indeed seems to be the case in the African continent where the progressive degradation of soil fertility seems to coincide with an increase in witchweed invasion. In developed countries, *Striga* spp. are not an agricultural problem, but the broomrapes are, despite the usually sufficient availability of phosphate fertilizers. Possibly, ample phosphate availability in developed world agriculture and consequently lower exudation of strigolactones by agricultural crops has resulted in selection pressure on broomrapes to respond to other chemicals as germination stimulants. Examples are the broomrape *Orobanche cumana* that parasitizes sunflower (*Helianthus annuus*) and germinates in response to dehydrocostuslactone (Joel et al., 2011) and the broomrape *Phelipanche ramosa* that is adapted to rapeseed (*Brassica napus*) and germinates in response to 2-phenylethyl isothiocyanate, a glucosinolate breakdown product (Auger et al., 2012; Figure 2). It is as yet unclear what—in vivo—the relative contribution is of the strigolactone(s) that these hosts also produce [sunflower also exudes the strigolactone heliolactone, which also induces germination in *O. cumana* (Ueno et al., 2014)] and these other germination stimulants to the infection success of the parasites. An intriguing question is also what adaptations evolved in the germination stimulant receptors of these parasites to enable the germination response to other chemical compounds. It is likely that there are other host-specific parasites that may have adapted to nonstrigolactone germination stimulants, giving them an exclusive niche as a parasite of a specific host. However, in broomrape hosts pea (*Pisum sativum*) and tomato (*Solanum lycopersicum*), for example, there is clear evidence that strigolactones are the main germination stimulant (Dor et al., 2011; Pavan et al., 2016).

**Box 1**

Analysis of germination stimulants. The discovery of germination stimulants started in the mid-1900s with the finding that root exudates of certain plant species induce the germination of seeds *of Striga hermonthica* (Brown et al., 1949) and reached a turning point 25 years later with the isolation and structural characterization of strigol (Cook et al., 1972). Ever since, bioassays and analytical chemistry have played an almost equally important role in studies on parasitic plant seed germination. For a bioassay, seeds of the parasitic plant under study are imbibed and incubated for 1 to 2 weeks at elevated temperatures to release their dormancy (a process referred to as pre-conditioning), which induces sensitivity to the germination stimulant(s) (Matusova et al., 2004; Figure 1). As a source of germination stimulants, (partially purified) root exudates, root extracts, and chemicals/standards can be used. These are applied to the pre-conditioned seeds which are subsequently incubated for a number of days after which germination is scored. A bioassay is suitable for screening of root exudates, for example of genotypes, for quantitative differences in germination stimulant activity. A bioassay does not allow conclusions on the chemical identity or number of different stimulant(s) present in the sample and the presence of inhibitors may cause a bias in the biological conclusions (Sato et al., 2003). Analytical tools, on the other hand, allow the detection and quantification of individual germination stimulants in a root exudate, such as for example the strigolactones that are analyzed using multiple reaction monitoring (MRM)-LC–MS/MS (Floková et al., 2020). For both assays, the instability of strigolactones combined with the very low produced amounts (in cotton in the order of 2–15 pg/plant/day; [Sato et al. 2005]) and the complexity of matrices make their isolation from root exudates and/or extracts and analysis difficult (Floková et al., 2020). The application of phosphorus starvation to the plants to be analyzed highly enhances the production and exudation of strigolactones, and therefore their detectability. Regardless of whether a root exudate or root extract is used for the analysis of strigolactones, a step of concentration and complexity reduction of the matrix using solid phase extraction is usually necessary (Floková et al., 2020).

Indeed, natural variation as well as induced mutations resulting in a decrease in strigolactone production have resulted in lower levels of germination and, hence, a certain degree of resistance against witchweeds and broomrapes in a number of crop species (Dor et al., 2011; Jamil et al., 2012a; Pavan et al., 2016). Also, a change in the strigolactone composition was shown to result in a lower induction of germination and, consequently, field resistance, as was shown for sorghum genotypes in which orobanchol is the predominant strigolactone instead of 5-deoxystrigol (Gobena et al., 2017). The important role of germination stimulants in the lifecycle of these parasitic plants has made them an interesting target for control (Box 3; Khan et al., 2008; Cardoso et al., 2011; Cimmino et al., 2014; Fernández-Aparicio et al., 2016; Masteling et al. 2019).

**Box 3**

Germination stimulants as target for parasitic weed management. Several parasitic plant species developed into agricultural weeds that cause tremendous yield losses in quite a number of crops. Rodenburg estimated that due to *Striga* infection in rice alone a yearly yield loss occurs of US $200 million (Rodenburg et al., 2016). Striga seed production per plant ranges between 5,000 and 84,000 seeds sometimes even reaching 200,000 (Hearne, 2009). The large number of seeds per plant and a long seed viability in the soil of up to 20 years contribute to the build-up of a seed bank and high infestation rates in the field. Different strategies to tackle the parasitic weed problem have been described including the use of herbicides, hand-pulling, etc. (Parker, 2012), but here we focus on methods that make use of or interfere with germination stimulants (Cardoso et al., 2011; Fernández-Aparicio et al., 2016). Germination stimulants are produced and exuded by true parasitic plant hosts but also by non-hosts. These nonhost plants have been widely used as intercrops and trap crops that induce massive germination but cannot be infected (intercrop) or are ploughed under after infection (trap crop) and therefore deplete the parasitic seed bank. The effect of intercrops seems to be enhanced by the release of allelopathic compounds that hamper the growth of or even kill the parasitic plant (Khan et al., 2008). A solution along the same line is the use of chemical analogs of the strigolactones that are applied to an infested field to induce suicidal germination (Samejima et al., 2016; Uraguchi et al., 2018; Kountche et al., 2019). Also, a range of plant- and microbe-produced compounds have been suggested to be suitable for this purpose (Cimmino et al., 2014; Masteling et al., 2019).

Reduction of parasitic plant seed germination could be another strategy for parasitic weed control. In pea, field screening for resistance against *Orobanche crenata* resulted in the identification of a partially resistant cultivar that exudes lower amounts of strigolactones (Pavan et al., 2016). The same holds true for rice where strong genetic variation for the amount of strigolactones exuded by the roots correlated with differences in *Striga* susceptibility (Jamil et al., 2012a). Low germination-based resistance can also be achieved through the type of strigolactone that is exuded. For instance, in sorghum the resistant genotype SRN39 was found to exude orobanchol instead of 5-deoxystrigol that is exuded by susceptible cultivars (Mohemed et al., 2016, 2018; Gobena et al., 2017).

### Strigolactones are the major class of germination stimulants

#### Strigolactones are a plant hormone

**Figure 3:**
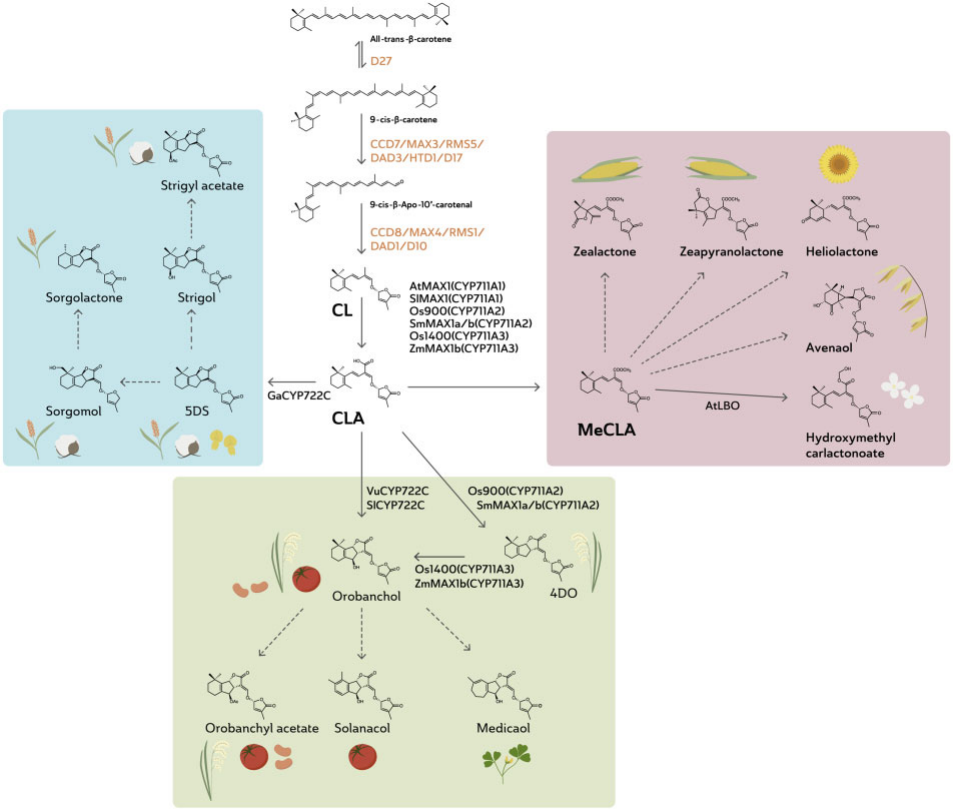
Schematic representation of strigolactone biosynthesis in a number of different plant species. Bold arrows indicate elucidated enzymatic steps; broken arrows indicated postulated biosynthetic steps. CL, carlactone; CLA, carlactonoic acid; MeCLA, methylcarlactonoate; 5DS, 5-deoxystrigol; 4DO, 4-deoxyorobanchol.

About 35 years after the identification of the first germination stimulant, strigol (Box 1), plant science was rocked by the discovery that the strigolactones are not just signaling molecules for parasitic plants, but that they also facilitate root colonization by arbuscular mycorrhizal (AM) fungi, as inducers of hyphal branching, a process preceding root colonization (Akiyama et al., 2005). Another 3 years later, two elusive phenomena—the branched/tillered phenotype of a series of *max/rms/dwarf* mutants in *Arabidopsis thaliana* (Arabidopsis), pea, and rice (*Oryza sativa*), respectively, and the genes that encode strigolactone biosynthesis—came together in back-to-back publications on the discovery that strigolactones are the long sought after hormone that controls branching in plants (Gomez-Roldan et al., 2008; Umehara et al., 2008). The authors showed that two carotenoid cleavage dioxygenases, CAROTENOID CLEAVAGE DIOXYGENASE 7 AND 8 (CCD7 and CCD8), are required for the biosynthesis of the branching hormone, strigolactone (Figure 3). What is still unclear after all these years, however, is which strigolactone-like/derived molecule is actually the internal hormone signal that controls branching. Mutations in *CCD7* and *CCD8* indeed result in a measurable decrease (or complete disappearance) in the level of strigolactones in root extracts and root exudates (Gomez-Roldan et al., 2008; Umehara et al., 2008; Vogel et al., 2010; Kohlen et al., 2012), but the evidence that these molecules are also active in the shoot is lacking (Xie et al., 2016). Despite this caveat in our knowledge, the two papers from 2008 triggered an avalanche of research on hormonal functions of the strigolactones. This research linked with the reported positive effect of phosphorus shortage on strigolactone production (Yoneyama et al., 2007b; Lopez-Raez et al., 2008), thought to stimulate root colonization by AM fungi under low phosphorus conditions (Bouwmeester et al., 2007). A number of studies showed that phosphorus starvation-induced strigolactone production is required for the adaptation of shoot (reduced branching/tillering) and root architecture (increased lateral root outgrowth) to these conditions (Koltai et al., 2010; Umehara et al., 2010; Kohlen et al., 2011; Ruyter-Spira et al., 2011). For other hormonal roles reported for the strigolactones, the link with phosphorus is less clear, but could play a role in the positive regulation by strigolactones of secondary stem growth, leaf senescence, and drought tolerance (Al-Babili and Bouwmeester, 2015; Yang et al., 2019). Finally, there are indications that strigolactones also affect the recruitment of other, possibly beneficial, microorganisms in the rhizosphere (Schlemper et al., 2017; Carvalhais et al., 2019).

These crucial hormonal and signaling roles for the host make strigolactones the ideal host presence signal for the parasitic plants, as even under very high selection pressure by parasitic plants, the host cannot evolve a complete absence of strigolactones. At first glance, the upregulation of strigolactone production under low phosphorus, however, does not seem an advantage for the parasitic plants, which results in higher parasitic plant seed germination and infection under conditions of low P availability and therefore reduced host vigor. It is unclear whether the parasites have also evolved mechanisms to compensate for that. On the other hand, since strigolactones are plant hormones, their upregulation under low phosphorus availability also has physiological consequences in the host. Branching/tillering is reduced, drought tolerance increased, leaf senescence stimulated, and root architecture changed through increased outgrowth of lateral roots (Snowden et al., 2005; Umehara et al., 2008; Ruyter-Spira et al., 2011; Haider et al., 2018). All these adaptations may be positive for the parasite as well. A large part of the physiological effects of strigolactones in the host is effectuated through an interaction with auxin, for example by affecting auxin transport (Van Rongen et al., 2019). Auxin plays an important role in the establishment of a connection between the parasite and host vasculature (Harb et al., 2004) and it seems that disturbance of normal auxin transport in strigolactone mutants results in higher susceptibility toward parasitic plant infection (Cheng et al., 2017).

#### Strigolactones display large structural diversity

So far, about 35 different strigolactones have been (tentatively) identified (Figure 2 andTable 1; Box 2; Cook et al., 1972; Hauck et al., 1992; Müller et al., 1992; Siame et al., 1993; Yokota et al., 1998; Xie et al., 2007; Abe et al., 2014; Kim et al., 2014; Ueno et al., 2014; Charnikhova et al., 2017, 2018). Different plant species usually exude different mixtures of several strigolactones. Many labs have investigated the structure–activity relationship of strigolactones and germination in different parasitic plant species. Eleven natural strigolactones were assessed for their germination-inducing activity in *Orobanche minor* (Kim et al., 2010). The monohydroxy-strigolactones (2′-*epi*-orobanchol, orobanchol, and sorgomol) were most active while the lipophilic strigolactones without oxygen-containing substituents (sorgolactone and 5-deoxystrigol) were the least active (Xie et al., 2007; Xie et al., 2008; Kim et al., 2010). For *S. hermonthica*, germination bioassays using structurally distinct strigolactones showed that strigol-type strigolactones induce higher germination than orobanchol-type (Nomura et al., 2013), while most of the broomrape germination stimulants are orobanchol-type strigolactones (Box 2). The so-called noncanonical strigolactones have germination stimulant activity toward broomrapes as well as *Striga* species. However, this observation is based on results with only a limited number of parasitic plant species.

**Box 2**

Discovery of strigolactone germination stimulants. The structure of the first strigolactone, strigol, was elucidated in 1972 (Cook et al., 1972; Table 1 andFigure 2). About 20 years later, sorgolactone was identified in the root exudates of sorghum as germination stimulant of *Striga asiatica* and *S. hermonthica* (Hauck et al., 1992; Table 1 andFigure 2). Alectrol (later renamed to orobanchyl acetate) was identified in the root exudate of cowpea and shown to be germination stimulant of *Alectra vogelii* and *Striga gesnerioides* (Müller et al., 1992). Strigol, initially discovered in nonhost cotton, was later also detected in the root exudates of several true *Striga* hosts (proso millet and maize and, in trace amounts, in sorghum; Siame et al. 1993). Later these hosts were shown to mainly produce other strigolactones, such as 5-deoxystrigol, sorgolactone, and sorgomol in sorghum and the noncanonical strigolactones, zealactone and zeapyranolactone in maize, and not or only very little strigol (Charnikhova et al., 2017, 2018; Mohemed et al., 2018; Table 1 andFigure 2). The name strigolactones was coined in 1995 to designate a, by then, small class of chemically similar compounds with *Striga* germination stimulant activity (Butler 1995). However, in 1998, it was shown that a compound also belonging to the strigolactones, orobanchol—isolated from the root exudate of red clover (*Trifolium pratense* L.)—induces germination of a broomrape (*Orobanche minor*; Yokota et al., 1998). A decade later, germination of another broomrape, *Phelipanche ramosa* L., was reported to be induced by solanacol, 2′-*epi*-orobanchol, and orobanchol, three strigolactones identified in the root exudate of *Nicotiana tabacum* L. (Xie et al., 2007; Figure 2). A range of additional strigolactones have since been identified and to date about 35 strigolactones have been (tentatively) identified (Table 1 andFigure 2). Intriguingly, most of the broomrape germination stimulants are orobanchol-type strigolactones (with α-oriented C-ring), while most *Striga* germination stimulants are strigol-type strigolactones (with β-oriented C-ring; Figure 2). The strigolactones can be further subdivided into canonical strigolactones with intact tricyclic lactone (ABC-rings) while noncanonical strigolactones—discovered in the past decade or so—only have the C-2′ *R*-configured D-ring in common with the canonical strigolactones (Abe et al., 2014; Ueno et al., 2014; Kim et al., 2014; Al-Babili and Bouwmeester, 2015; Charnikhova et al., 2017; Figure 2). They have germination stimulant activity towards broomrapes as well as *Striga* spp.

**Table 1:** Overview of all strigolactones identified to date

Type	Name	Plant species^a^	References^b^
Strigol-type SLs	5-Deoxystrigol	*Lotus japonicus, Sorghum bicolor, Pennisetum typhoideum, Nicotiana tabacum*	(Akiyama et al., 2005; Awad et al., 2006; Xie et al., 2013)
	Strigol	*Gossypium hirsutum, Sorghum bicolor, Panicum miliaceum*	(Cook et al., 1966; Siame et al., 1993)
	Strigyl acetate	*Gossypium hirsutum*	(Cook et al., 1966)
	Sorgomol	*Sorghum bicolor*	(Xie et al., 2008)
	Sorgolactone	*Sorghum bicolor*	(Hauck et al., 1992)
	Strigone	*Houttuynia cordata*	(Kisugi et al., 2013)
	4α-Hydroxy-5-deoxystrigol/ *Ent*-2'-*epi*-orobanchol	*Vigna unguiculate, Trifolium pratense, Nicotiana tabacum*	(Ueno et al., 2011; Xie et al., 2013)
	4α-Acetoxy-5-deoxystrigol/ *Ent*-2'-*epi*-orobanchyl acetate	*Vigna unguiculate, Trifolium pratense, Nicotiana tabacum*	(Ueno et al., 2011; Xie et al., 2013)
	7β-Hydroxy-5-deoxystrigol	*Houttuynia cordata*	(Yoneyama et al., 2018b)
Orobanchol-type SLs	4-Deoxyorobanchol	*Oryza sativa, Nicotiana tabacum*	(Umehara et al., 2008; Xie et al., 2013)
	Orobanchol	Trifolium pratense, *Oryza sativa, Nicotiana tabacum, Sorghum bicolor, Solanum lycopersicum, Medicago sativa, Pisum sativum*	(Yokota et al., 1998; Awad et al., 2006; Xie et al., 2007; López‐Ráez et al., 2008; Yoneyama et al., 2008; Jamil et al., 2011)
	Orobanchyl acetate	*Vigna unguiculata, Trifolium pratense, Pisum sativum, Glycine max*	(Müller et al., 1992; Yokota et al., 1998; Xie et al., 2008; Yoneyama et al., 2008)
	Solanacol	*Nicotiana tabacum, Solanum lycopersicum*	(Xie et al., 2007; López‐Ráez et al., 2008)
	Solanacyl acetate	*Nicotiana tabacum*	(Xie et al., 2007, 2013)
	Fabacol	*Pisum sativum*	(Xie et al., 2009a)
	Fabacyl acetate	*Pisum sativum*	(Xie et al., 2009a)
	7-Oxoorobanchol	*Linum usitatissimum, Cucumis sativus*	(Xie et al., 2009b; Khetkam et al., 2014)
	7-Oxoorobanchyl acetate	*Linum usitatissimum, Cucumis sativus*	(Xie et al., 2009b; Khetkam et al., 2014)
	7α-Hydroxyorobanchol	*Cucumis sativus*	(Khetkam et al., 2014)
	7α-Hydroxyorobanchyl acetate	*Cucumis sativus*	(Khetkam et al., 2014)
	7β-Hydroxyorobanchol	*Cucumis sativus*	(Khetkam et al., 2014)
	7β-Hydroxyorobanchyl acetate	*Cucumis sativus*	(Khetkam et al., 2014)
	Medicaol	*Medicago truncatula*	(Tokunaga et al., 2015)
Noncanonical SLs	Zealactone	*Zea mays*	(Charnikhova et al., 2017; Xie et al., 2017)
	Zeapyranolactone	*Zea mays*	(Charnikhova et al., 2018)
	Avenaol	*Avena strigosa*	(Kim et al., 2014)
	Heliolactone	*Helianthus annuus*	(Ueno et al., 2014)
	Carlactone	*Arabidopsis thaliana, Oryza sativa*	(Seto et al., 2014)
	Carlactonoic acid	*Arabidopsis thaliana, Oryza sativa, Selaginella moellendorffii, Populus trichocarpa*	(Abe et al., 2014; Yoneyama et al., 2018a)
	Methyl carlactonoate	*Arabidopsis thaliana*	(Abe et al., 2014; Seto et al., 2014)
	3-Hydroxycarlactone	*Oryza sativa*	(Baz et al., 2018)
	Lotuslactone	*Lotus japonicus*	(Xie et al., 2019)
	Putative SL in rice	*Oryza sativa*	(Yoneyama et al., 2018b)
	Putative SL in black oat	*Avena strigosa*	(Yoneyama et al., 2018b)

a For some SLs (5-deoxystrigol, 4-deoxyorobanchol, orobanchol, orobanchyl acetate) only representative plant species with more than two reports are shown.

b Only the first report per plant species is shown here.

The strigolactone concentrations required for the induction of germination of parasitic plants vary from pM to µM, depending on the strigolactone, likely as a result of differences in the parasitic plant germination stimulant receptor or downstream signaling, possibly reflecting the co-evolution of the parasitic plant species with its hosts (Kim et al., 2010; Kisugi et al., 2013; Yoneyama et al., 2018b). It is tempting to speculate that parasitic plants and/or pathogenic microorganisms [that have also been suggested to be attracted by strigolactones (López-Ráez et al., 2017)] have exerted a selective pressure driving changes in strigolactone structure (while keeping the specificity for symbiotic organisms such as the AM fungi), which has resulted in the large structural diversity in the strigolactones as we know it today. Parasitic plants, in turn, seem to have evolved mechanisms to perceive the different strigolactones, resulting in the large number of receptor copies found in the genomes of parasitic plants. With more in-depth studies, new natural strigolactones will be discovered and their structures determined. These will be tested with various parasitic plant species, and this should gradually improve our understanding of the relationship between strigolactone structure and activity as germination stimulants.

### The biosynthesis of strigolactones is only partially elucidated

Strigolactones were initially considered to be sesquiterpene lactones (Butler, 1995; Yokota et al., 1998). However, root exudates from plants treated with the carotenoid biosynthesis inhibitor fluridone and maize mutants deficient in carotenoid biosynthesis induced lower *Striga* seed germination than the untreated and wild-type controls, respectively, indicating that the maize germination stimulants—which were assumed to be strigolactones—derive from the carotenoids (Matusova et al., 2005). After the discovery that strigolactones are also plant hormones that control branching/tillering, forward genetics analyses of more branching/high tillering mutants helped the community to start to unravel strigolactone biosynthesis and signaling. Identification of the genes underlying these mutations and their functional characterization resulted in the discovery of several key strigolactone biosynthetic genes: *β-CAROTENE ISOMERASE*, *D27*; *CCD7* (*MAX3*/*RMS5*/*DAD3*/*HTD1*/*D17*), and *CCD8* (*MAX4*/*RMS1*/*DAD1*/*D10*; Morris et al., 2001; Stirnberg et al., 2002; Sorefan et al., 2003; Booker et al., 2004, 2005; Foo et al., 2005; Ishikawa et al., 2005; Snowden et al., 2005; Arite et al., 2007; Simons et al., 2007; Drummond et al., 2009; Lin et al., 2009; Drummond et al., 2012; Figure 3). *D27* encodes a β-carotene isomerase, converting all-*trans*-β-carotene into 9-*cis*-β-carotene (Alder et al., 2012; Bruno and Al-Babili, 2016; Abuauf et al., 2018). 9-*Cis*-β-carotene is cleaved by CCD7 into 9-*cis*-β-apo-10-carotenal and β-ionone (Alder et al., 2012). The former is further converted by CCD8 into carlactone (Alder et al., 2012; Al-Babili and Bouwmeester, 2015). It is currently assumed that carlactone is the precursor for all strigolactones.

The identification of biosynthetic steps downstream of carlactone is more challenging as mutants do not display a (clear) branching/tillering phenotype. To a certain extent, this also holds true for the CYP711AV1 cytochrome P450, *MORE AXILLARY GROWTH1, MAX1*. *MAX1* was discovered through forward genetics in Arabidopsis, which only has one copy of the gene (Booker et al., 2005), but no *max1* mutants are known for rice likely because it has four or five homologs (Zhang et al., 2014). Biochemical characterization of MAX1 homologs from various plant species showed they can be classified into three types (Seto et al., 2014; Zhang et al., 2014, 2018; Yoneyama et al., 2018a; Figure 3). The A1 MAX1s, including AtMAX1 and its homologs from tomato and poplar, convert carlactone into carlactonoic acid. The A2 MAX1s (rice Os900 and the *Selaginella* SmMAX1a/b) produce 4-deoxyorobanchol from carlactone. The A3-type MAX1s display both activities, and include rice Os1400 and maize ZmMAX1b.

So far, very few other downstream enzymes involved in the diversification of strigolactone biosynthesis in other plant species have been identified. In Arabidopsis, carlactonoic acid is methylated into methyl carlactonoate by an as yet unknown methyl transferase (Abe et al., 2014; Seto et al., 2014). Using transcriptomics and co-expression analysis, *LATERAL BRANCHING OXIDOREDUCTASE*, *LBO*, was identified and shown to reversibly convert methyl carlactonoate into 1′-hydroxymethyl carlactonoate (1′-HO-MeCLA; Brewer et al., 2016; Yoneyama et al., 2020; Figure 3). Through RNA-seq and co-expression gene network analysis, a cytochrome P450, CYP722C, was identified in cowpea (*Vigna unguiculata*) and tomato that catalyses the conversion of carlactonoic acid to orobanchol (Wakabayashi et al., 2019; Figure 3). Intriguingly, the homolog from cotton (*Gossypium arboreum*), GaCYP722C, catalyzes the formation of the strigol-type strigolactone, 5-deoxystrigol, from carlactonoic acid (Wakabayashi et al., 2020) similar to LjCYP722C from *Lotus japonicus* (Mori et al., 2020). Taken together, this suggests that the CYP722C family is essential for the production of canonical strigolactones in dicots (Wakabayashi et al., 2020). To gain a better understanding of the importance of the structural diversity in the strigolactones, elucidation of their biosynthesis is imperative.

#### Strigolactone production is regulated by environmental conditions

The evolution of a dual role for strigolactones as plant hormones and rhizosphere signaling molecules has resulted in quite a complex regulation of their biosynthesis, on the one hand, to control development, and on the other hand, to mediate symbiosis, both in response to environmental conditions and in crosstalk with other plant hormones such as auxin, cytokinin, ABA, ethylene, and gibberellins (Cheng et al., 2013; Al-Babili and Bouwmeester, 2015). As described above, under phosphorus shortage, plants secrete strigolactones to attract AM fungi (Gutjahr, 2014). Indeed, strigolactone biosynthesis and production are induced by phosphorus (and nitrogen) shortage in many plant species, including Arabidopsis, rice, maize, sorghum, red clover, tomato, and sunflower (Yoneyama et al., 2007a, 2007b; Lopez-Raez et al., 2008; Umehara et al., 2010; Jamil et al. 2011, 2012b, 2013; Kohlen et al., 2011; Ueno et al., 2014; Ravazzolo et al., 2019). The elevated strigolactone production under nutrient deficiency mainly results from the upregulation of transcription of the strigolactone biosynthetic genes (*D27*, *CCD7*, *CCD8*, *MAX1*), as observed in rice, *Medicago truncatula*, and tomato (Bonneau et al., 2013; Sun et al., 2014; Wen et al., 2016; Wang et al., in press). In contrast, transcript levels of strigolactone biosynthesis and transporter genes are down-regulated with sufficient phosphorus supply, as shown for *CCD8* and the strigolactone transporter *PhPDR1* in petunia (Breuillin et al., 2010; Kretzschmar et al., 2012). Interestingly, strigolactone exudation by *Physcomitrella patens* was also shown to be increased by phosphorus starvation, suggesting that the role of strigolactones and their regulation by phosphorus availability in plants are evolutionarily conserved (Decker et al., 2017).

#### Strigolactones are perceived by several different receptors

Forward genetics studies in Arabidopsis, rice, and petunia (*Petunia hybrida*) have identified an α/β-fold hydrolases, DWARF14 (D14), as the strigolactone hormone-receptor in angiosperms (Arite et al., 2009; Gaiji et al., 2012; Hamiaux et al., 2012; Waters et al., 2012). In parallel, a homolog of *D14*, *HYPOSENSITIVE TO LIGHT (HTL)* or *KARRIKIN INSENSITIVE2 (KAI2)*, was discovered as the receptor of karrikins (KARs), butenolide compounds present in smoke that stimulate the seed germination of fire-succession land plants (Sun and Ni, 2011; Waters et al., 2012). D14 is only present in seed plants but KAI2 is present in algae, mosses, and all vascular land plants (Lopez-Obando et al., 2016), suggesting that KAI2 perceives an unknown ligand, coined the KAI2-Ligand (KL), and that this predates KAR perception (Conn and Nelson, 2016). Most likely, *D14* evolved via duplication from the ancestral *KAI2* (Delaux et al., 2012; Waters et al., 2012; Conn and Nelson, 2016). Both D14 and KAI2 have the catalytic triad residues, Ser95–Asp217–His246, capable of hydrolyzing butenolide substrates (Nakamura et al., 2013; De Saint Germain et al., 2016; Yao et al., 2016a). However, it is under debate whether this catalytic activity is required for signaling (Shabek et al., 2018; Seto et al., 2019; Yao and Waters, 2020). Genome/transcriptome analysis of parasitic plants showed that they have one *D14* (Das et al., 2015), which likely encodes the receptor of endogenous strigolactones of the parasites (Xu et al., 2018). Two groundbreaking studies showed that parasitic Orobanchaceae have multiple *KAI2/HTL* copies that encode the receptor for the perception of exogenous, host strigolactones (Conn et al., 2015; Toh et al., 2015; and Nelson et al., this issue). The work showed that in these parasites *KAI2/HTL* duplicated, and neo-functionalized for the detection of strigolactones, resulting in a clade that contains 12 *S. hermonthica HTLs* (*ShHTL4-11*) as well as four to six copies in broomrapes such as *O. cumana, Phelipanche aegyptiaca*, *Orobanche cernua*, and *O. minor* (based on transcriptomes not genome sequences; Conn et al., 2015).

**Figure 4:**
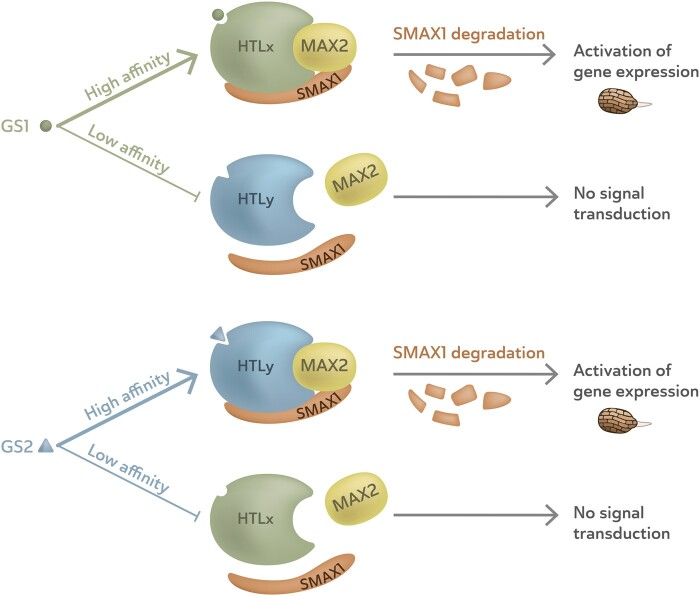
Schematic representation of the perception of germination stimulants through the HTL receptors and the effect of differences in affinity. Germination stimulants (GS1 and 2) bind to an HTL that then recruits MAX2. The activated complex degrades downstream repressor, SMAX, which results in de-repression of gene expression and induces germination. In case of low affinity of the HTL present in the seed for the host germination stimulant, signal transduction and germination do not occur. Host-specific germination in root parasitic plants could be driven by affinity differences between HTLs for host-produced germination stimulants.

In addition to the receptor HTL, other components are required for the induction of germination (Figure 4). Intriguingly, both D14 and KAI2/HTL require the same F-box protein MORE AXILLARY GROWTH2 (MAX2) for signal transduction (Stirnberg et al., 2002; Nelson et al., 2011; Waters et al., 2012). However, interaction of D14 or KAI2/HTL with MAX2 results in different physiological responses as their downstream signaling partners are different (Nelson et al., 2011; Chevalier et al., 2014; Waters et al., 2015; Yao and Waters, 2020). MAX2 activates the ubiquitination of specific target proteins, which belong to the SUPPRESSOR OF MAX2-LIKE family (Figure 4). Since *MAX2* is present in parasitic plant genomes, and *ShMAX2* can rescue Arabidopsis *max2*, it is highly likely that perception of strigolactones in parasitic plants is also MAX2 dependent (Liu et al., 2014; Conn et al., 2015; Bunsick et al., 2020). Strigolactone binding to HTL induces a conformational change that facilitates MAX2 binding. Upon MAX2 binding to HTL, the proteasome-mediated degradation of repressor SMAX1 is activated, which eventually results in seed germination (Bunsick et al., 2020; Khosla et al., 2020; Figure 4).

Protein crystallography and computational modeling have shown that the parasite-specific clade HTLs have larger binding pockets than KAI2 (Xu et al., 2018). Hence, these HTLs can interact with the bulkier strigolactones as shown by Arabidopsis *kai2* mutant complementation studies and competition assays with the fluorescent analog substrate yoshimulactone green (Conn et al., 2015; Toh et al., 2015; Tsuchiya et al., 2015; also see Nelson et al., this issue). Intriguingly, these HTLs have lost responsiveness to (the less bulky) KAR, the high-affinity substrate of KAI2 (Xu et al., 2018).

Although the divergent clade *HTLs* across different parasitic plant species have more than 50% sequence identity, their affinity toward different strigolactones varies considerably (Conn et al., 2015; Xu et al., 2018; Zhang et al., 2020). The main structural variation between HTLs occurs in the V-shape helical cap domain, at the entrance of the binding pocket, which is formed by helices αD1 and αD2. Changes in the amino acid composition of αD1 affect its position relative to αD2, thus creating binding pockets with different volumes (Xu et al., 2018). Several studies have suggested that the main factor determining the affinity of the HTLs for specific strigolactones is the architecture and size of this binding pocket (Xu et al., 2018; Zhang et al., 2020). For example, ShHTL7 has evolved to have less bulky amino acid residues, which results in enlargement of the binding pocket. Indeed, upon complementation of the Arabidopsis *kai2* mutant with *ShHTL7*, germination of Arabidopsis could be triggered by pM concentrations of GR24 (Toh et al., 2015). Particularly, ShHTL4,6-10 display a high affinity to natural strigolactones (Toh et al., 2015; Tsuchiya et al., 2015; Zhang et al., 2020). It is tempting to speculate that the differences in affinity for different strigolactones between the HTLs are the underlying mechanism for host specificity in germination (Figure 4).

### Germination stimulants play a role in host specificity

In vitro assays have shown that different parasitic plant species mount distinct responses to different germination stimulants. For instance, *O. cumana* has a higher germination rate with strigol than fabacyl acetate, while *O. hedera* showed a higher response to fabacyl acetate than strigol (Fernández-Aparicio et al., 2010). Something similar holds true for the witchweeds. *S. hermonthica* ecotypes collected from maize and sorghum responded differentially to the exudates of maize (*Zea mays*), cowpea (*Vigna unguiculata*), and the synthetic germination stimulant GR24 (Matusova and Bouwmeester, 2006). The best in vivo example of the importance of germination stimulants in determining host specificity is the *Striga* resistance of the sorghum (*Sorghum bicolor*) genotypes that produces orobanchol instead of the 5-deoxystrigol that is produced by susceptible genotypes (Mohemed et al., 2016; Gobena et al., 2017; Mohemed et al., 2018). Intriguingly, pearl millet (*Pennisetum glaucum*), which has been shown to produce orobanchol-type strigolactones, and maize, producing noncanonical strigolactones, are also severely infested by *S. hermonthica*. Two ecotypes of *S. hermonthica* collected from sorghum and millet, displayed very different germination when exposed to a sorghum root exudate (Awadallah and Dafaallah, 2020) and vice versa, germination of *S. hermonthica* collected from sorghum was much lower with pearl millet root exudate (Nasreldin, 2018). These differences become even more puzzling by the observation that in regions dedicated to the cultivation of sorghum, *S. hermonthica* displayed rapid adaptation upon introduction of new host species that were initially not infected. Curiously, species such as barley (*Hordeum vulgare*), wheat (*Triticum aestivum*), and teff (Eragrostis tef)—which are considered nonhost species for *Striga*—have shown severe infestations over time (Ejeta, 2007). This suggests that *Striga* can rapidly adapt to a new host, including to its germination stimulants. The mechanism underlying this adaptation has not been elucidated. Based on amplified fragment length polymorphism analysis, *S. asiatica* ecotypes adapted to different hosts were indeed genetically distinguishable (Botanga et al., 2002), albeit a study conducted in Ethiopia did not find this for *S. hermonthica* (Welsh, 2011).

It is still an enigma whether strigolactone receptors, encoded by the *HTLs*, play a role in this host specificity and adaptation and, if so, what the underlying mechanisms are. A transcriptomics study on the effect of the host on *S. hermonthica* gene expression demonstrated an influence of the host on parasite gene expression, including genes involved in defense, pathogenesis, and plant hormone response (Lopez et al., 2019). As pointed out by the authors, these observations made during the vegetative stage of the parasite indicate the importance of studies to investigate the host–parasite interaction beyond the haustorial connection.

In the witchweeds, strigolactones are the main germination stimulants. However, in the broomrapes, there are several examples of other, sometimes structurally similar, compounds that act as germination stimulants in, so it seems, very specific host–parasite combinations. These compounds sometimes resemble the strigolactones, but some are quite structurally different, which raises the question if these germination stimulants are perceived by an HTL receptor and if so, how this receptor evolved affinity for such different molecules. A striking example is the response of a *B. napus P. ramosa* ecotype that responds to 2-phenylethyl isothiocyanate (Matusova and Bouwmeester, 2006; Auger et al., 2012; Figure 2). Strigolactones have not yet been identified in the root exudate of *B. napus* (Auger et al., 2012) possibly because the Brassicaceae are not a host to AM fungi. Also intriguing is the host specificity of *O. cumana* that parasitizes sunflower and responds to the sesquiterpene lactones dehydrocostus lactone, costunolide, tomentosin, and 8-epixanthatin as germination stimulants (Joel et al., 2011; Raupp and Spring, 2013) but also to the strigolactone heliolactone (Ueno et al., 2014; Figure 2). Interestingly, seeds of *P. ramosa* do not respond to sesquiterpene lactones showing the specificity of the *O. cumana* receptor, possibly HTL, for these compounds. A rigorous evaluation of the affinity of the HTLs of these different parasitic plant species for these (putative) germination stimulants from other chemical classes should provide more insight into the remarkable examples of the evolution of germination stimulant-mediated host specificity.

## Concluding remarks

Research done in the past 10–20 years has greatly improved our knowledge on the biological relevance of germination stimulants, for both root parasitic plants as well as their hosts. This particularly concerns our knowledge on the diverse roles of strigolactones as well as their biosynthesis and perception, but also the discovery of alternative, nonstrigolactone, germination stimulants. So far the involvement of germination stimulants has only been demonstrated in the broomrapes and witchweeds. Facultative parasites from the Orobanchaceae and parasitic plants from other families are assumed not to use any host presence cues, such as germination stimulants. However, it would seem that the response to host cues confines an evolutionary advantage also for facultative parasites. The expansion of *KAI2/HTLs* that now was also demonstrated for the facultative parasite *Phtheirospermum japonicum* (Conn et al., 2015) suggests we may have overlooked such cues and more careful germination assays should show if this is true (see Outstanding questions box).

What we have learned from the strigolactones is that evolution in a parasite of a developmental dependence on a host signal must make use of an essential host molecule that cannot easily disappear under selection pressure. The enormous structural diversity that we are uncovering in the strigolactones suggests that this selection pressure does exist, and is resulting in an arms race-like diversification in chemical structure to secure specificity in beneficial signaling relations and exclude pathogens. Why, however, most plant species produce blends of different strigolactones remains a conundrum. Possibly they are involved in additional underground signaling relations that we have not identified yet with different strigolactones mediating different relations. Examples of the latter could be the role of strigolactones as signals for other beneficial microorganisms such as phosphate-solubilizing and nitrogen-fixing bacteria (see Outstanding questions box).

So indeed, the obvious biological importance for the host makes the strigolactones a reliable germination stimulant, but an intriguing question remains how the parasites have evolved mechanisms to deal with low phosphorus availability, as under these conditions, germination-stimulant production and therefore infection is high, as is especially clear in the witchweeds. As discussed, in the broomrapes, selection for other germination stimulants seems to have occurred. This possibly removed the link between the germination stimulant and low phosphorus, which could be a selective advantage, provided that the new germination stimulant is also essential for the host and cannot easily be selected against. The sunflower and rapeseed germination stimulants for *O. cumana* and *P. ramosa* possibly fulfill these requirements because they represent secondary metabolites that may play an essential role in pathogen or insect protection of the host. The evolution of new germination stimulants requires also evolution of new receptors in the parasites. The expansion of the HTL receptor clade in parasitic plants represents an intriguing science field. Further biochemical characterization of the receptors and what determines their expression, as well as the possibility to manipulate their expression in a model parasite will allow us to further unravel the role of these receptors in the interaction of hosts and parasites, including the extent to which they contribute to host specificity (see Outstanding questions box).

## Funding

This work was supported by the European Research Council (ERC) Advanced grant CHEMCOMRHIZO (670211; to H.J.B., M.R., and L.D.), a China Scholarship Council (CSC) scholarship (to C.L.), Bill & Melinda Gates Foundation grant Promise (OPP1082853; to H.J.B. and B.T.) and Marie Curie fellowship NEMHATCH (793795; to L.D.).

*Conflict of interest statement*. None declared.

**Advances**

- In the past decade, the roles of germination stimulants and their perception in regulating the lifecycle of orobanchaceous root parasitic plants have been uncovered.
- Over 35 different strigolactones have been discovered in a large array of plant species and shown to induce the germination of a range of orobanchaceous parasitic plant species.
- Strigolactones are allelochemical cues for (symbiotic) microorganisms and plant hormones that regulate several developmental processes.
- The germination stimulant receptor in parasitic plants evolved from an ancestral homolog of the strigolactone hormone receptor.
- Strigolactones exhibit large structural diversity; however, the biological relevance of this diversity is unclear.

## Notes

H.B. outlined the review; H.B., C.L., B.T., M.R., and L.D. wrote the review; H.B. aligned and integrated the different sections.

The author responsible for distribution of materials integral to the findings presented in this article in accordance with the policy described in the Instructions for Authors (https://academic.oup.com/plphys/pages/general-instructions) is: Harro Bouwmeester (h.j.bouwmeester@uva.nl).
